# CIDP mimics: a case series

**DOI:** 10.1186/s12883-021-02118-7

**Published:** 2021-02-28

**Authors:** Orly Moshe-Lilie, Erik Ensrud, Thomas Ragole, Chahin Nizar, Diana Dimitrova, Chafic Karam

**Affiliations:** 1 Department of Neurology, Oregon Health & Science University, Portland, OR USA; 2 Department of Neurology, Hospital of the University of Pennsylvania, 3400 Spruce St., 3 West Gates, Philadelphia, PA 19104 USA

**Keywords:** CIDP, CIDP mimics, POEMS, CANOMAD, Neurolymphomatosis

## Abstract

**Background:**

To report our experience with a group of patients referred for refractory CIDP who fulfilled “definite” electrodiagnostic EFNS criteria for CIDP but were found to have an alternate diagnosis.

**Methods:**

Patients who were seen between 2017 and 2019 for refractory CIDP that fulfilled “definite” electrodiagnostic ENFS criteria for CIDP, but had an alternate diagnosis, were included. Patients who correctly had CIDP, anti MAG neuropathy, or MMN with conduction block, were excluded from the study. Demographics, clinical and electrophysiological characteristics, pertinent workup, final alternate diagnoses, and outcomes were collected.

**Results:**

Seven patients were included: POEMS (*n* = 5), CANOMAD (*n* = 1), and neurolymphomatosis (n = 1). Most patients reported neuropathic pain and leg swelling (*n* = 6) or significant weight loss (*n* = 4). All patients had a monoclonal protein, and most patients who were tested had an elevated VEGF and CSF cyto-albuminologic dissociation. Electrophysiology showed pronounced intermediate more than distal demyelination, and axonal loss in the lower extremities. Response to steroids or IVIG varied, but some patients did respond to these treatments, especially early in the disease.

**Conclusion:**

Pain, systemic symptoms, suggestive electrophysiological findings, and/or a serum monoclonal protein should raise suspicion for CIDP mimics. Initial response to steroids or IVIG, over reliance on CSF, and electrophysiology findings can all be misleading.

## Background

Over a dozen guidelines and expert consensus exist to aid with the adequate diagnosis of chronic inflammatory demyelinating polyneuropathy (CIDP). While some of these guidelines, such as the “European Federation of Neurological Societies/Peripheral Nerve Society (ENFS/PNS) Guideline on management of chronic inflammatory demyelinating polyradiculoneuropathy”, are highly sensitive and specific for CIDP [1, 2], misdiagnosis remains common [3, 4]. Among the many sources of misdiagnosis, the lack of adherence to published guidelines is prominent [1, 2]. For example, in a series of patients misdiagnosed with CIDP, more than 85% of patients did not fulfill the definite EFNS/PNS criteria [3].

While following the EFNS/PNS CIDP guidelines for CIDP would dramatically reduce the rate of misdiagnosis, there remains a small number of patients who fullfill these criteria for CIDP but may have an alternative diagnosis (“true CIDP mimics”) [5–8]. These patients may experience delay in appropriate diagnosis and initiation of adequate therapy resulting in significant disability and morbidity [6]. Whereas most studies have focused on “over” diagnosis of CIDP, mainly in patients not fulfilling ENFS/PNS criteria, we aimed to discuss a group of patients who were referred for “refractory CIDP” and fulfilled “definite” electrophysiological ENFS/PNS criteria for CIDP, but had an alternate diagnosis.

## Methods

The Oregon Health & Science University Institutional Review Board approved this study. We reviewed the charts of all patients seen by our neuromuscular group between 2017 and 2019 who were referred for CIDP management and fulfilled definite European ENFS CIDP criteria. We excluded patients who were eventually diagnosed with CIDP, anti MAG neuropathy, or multifocal motor neuropathy with conduction block. We included patients who fulfilled definite European ENFS CIDP criteria but eventually had an alternate diagnosis. We collected and summarized data on demographics (age and gender), symptomatic onset and manifestations (neuropathic pain, autonomic dysfunction and syncope, weight loss), findings on clinical examination (early onset muscle atrophy, tremor, ataxia, ophthalmoplegia, organomegaly, edema, skin changes), pertinent laboratory findings (CSF protein, monoclonal protein and VEGF levels), any pertinent imaging findings, and noted initial and final alternate diagnoses as well as outcomes. Finally, electrophysiologic characteristics were summarized, and terminal latency index (TLI) was calculated using the following formula: TLI = terminal distance (mm)/(distal latency (ms) × conduction velocity (m/s)). The datasets used and/or analyzed during the current study available from the corresponding author on reasonable request.

## Results

Seven patients were diagnosed with CIDP and fulfilled “definite” electrophysiological ENFS/PNS criteria for CIDP, but subsequently were found to have one of the following alternate diagnoses: POEMS syndrome (*n* = 5), CANOMAD (*n* = 1), and neurolymphomatosis (*n* = 1). All of these patients were initially seen and diagnosed with CIDP by outside neuromuscular experts. The demographics, clinical characteristics of these patients and data collected are summarized in Table 1.

**Table 1:** Patients referred for “refractory CIDP” who met “definite” CIDP criteria but had alternate diagnosis

	POEMS	CANOMAD	Neurolymphomatosis
**Number of patients, N**	5	1	1
**Age, median (range)**	52 (33–66)	61	77
**Sex**			
**M**	4	1	0
**F**	1	0	1
**Onset**			
**Acute**	1	0	0
**Subacute**	3	0	1
**Insidious**	1	1	0
**Weakness**			
**Uppers**	5	1	1
**Lowers**	5	1	1
**Diminished sensation**			
**Uppers**	4	1	1
**Lowers**	4	1	1
**Pain**	4	1	1
**Ataxia**	0	1	0
**Tremor**	0	1	0
**Ophthalmoplegia**	0	1	0
**Edema**	5	0	1
**Autonomic dysfunction**	2	0	0
**Diabetes**	0	0	0
**Weight loss**	3	0	1
**Early onset atrophy**	2	0	0
**Skin changes**	4	0	0
**Sclerotic bony lesions**	3	0	0
**CSF cyto-albuminologic dissociation, N (range)**	3 (90–144)	1 (84)	0 (34)
**Monoclonal protein, N**			
**Lamda**	5	0	1
**Kappa**	0	1	0
**VEGF elevation, N (range)**	4 (151–771)	N/A	0
**ANA positivity, N (ratio)**	1 (1:320)	N/A	0
**TLI**			
**Median (wrist-elbow)**	0.67 (0.58–0.79)	0.47	0.52
**Ulnar (wrist-below elbow)**	0.67 (0.57–0.76)	0.48	0.59

Presentation was acute in one patient, subacute in four patients, and insidious in two patients. All patients had proximal and distal motor weakness and diminished sensation in all extremities. The most common symptom in these patients (six of seven) was severe neuropathic pain. In the POEMS patients, leg swelling was universal (five of five). Skin changes (four of five) and pronounced weight loss, ranging from 50 to 200 lbs. (three of five), were also prominent clinical features. Less common features within this patient group were early onset muscle atrophy, autonomic dysfunction with reported syncope, and sclerotic bone lesions. The patient with CANOMAD had significant ataxia, tremor and ophthalmoplegia, and had IgG disialosyl GD1b antibodies. Five of seven patients had CSF examination. Four of five patients had cyto-albuminologic dissociation and were found to have protein levels ranging from 84 to 144 mg/dL. All patients had a monoclonal protein on serum immunofixation; IgG lambda monoclonal protein of 69.4 mg/L (normal range 5.7–26.3 mg/L) in the neurolymphomatosis patient; all POEMS patients had monoclonal lambda protein (IgA lambda (20.7–61/99 mg/L) in 3 patients, IgG lambda (40.25–49.1 mg/L) in 2 patients). Four of 5 POEMS patients had elevated VEGF level, ranging from 151 to 771 pg/mL (normal value < 96.2 pg/mL), one had normal VEGF level while on steroids.

On electrophysiologic testing, six of seven patients had significantly high TLI, most pronounced within POEMS patients (see table) when compared to median ranges of TLI reported for CIDP patients (0.30 (0.07–0.69) for median nerve TLI and 0.42 (0.12–0.70) for ulnar nerve TLI) [9, 10]. All of our patients had some degree of motor conduction block; two had greater than 50% amplitude reduction of the proximal negative peak compound motor action potential relative to distal, and three had a drop in the amplitude ranging from 37 to 48%.

## Discussion

Despite the raised awareness of CIDP mimics and several guidelines for CIDP diagnosis, there are still some patients who are misdiagnosed as having CIDP by neuromuscular experts. The over reliance on electrodiagnostic studies, even if they meet definite demyelination, and the over reliance on CSF findings as well as initial response to treatment, can be misleading [11]. These “true” CIDP mimics (in contrast to patients misdiagnosed with CIDP in general who most do not fit the ENFS CIDP criteria), pose a significant diagnostic challenge to neurologists, even for those with neuromuscular expertise. The clinical “red flags” seen here such as the presence of pain, systemic symptoms such as swelling, various skin changes (from darkened or tanned-like skin appearance to papules, acne-like bumps or patches of erythema) and weight loss should push the physician to evaluate for other causes of neuropathy, even when there is definite demyelination on electrodiagnostic testing. A monoclonal protein, especially of IgM subtype or lambda subtype, should also raise suspicion of an alternate diagnosis. Another “red flag” to consider is demyelination in intermediate more than distal nerve segments based on high TLI, which is more frequently seen in POEMS patients in contrast to CIDP. In POEMS patients demyelination usually affects the roots and distal nerves [7, 10]. As is well known, albumin-cytologic dissociation on CSF examination is not specific for CIDP and should not be relied on for diagnosis of CIDP.

Among CIDP mimics, POEMS syndrome may be the mostly frequently encountered. While it may be easy to suspect POEMS when there are all the typical features such as skin changes, endocrinopathy, organomegaly, etc., some patients, especially early in their disease, may present with pure neuropathic features [7, 10]. These patients may be difficult to recognize without screening for monoclonal protein. However, POEMS can be suspected if careful attention is paid to electrophysiological findings. As noted, the clues for possibility of POEMS are the following [1]: high TLI, which indicates more prominent intermediate than distal demyelination [2]; axonal loss in the lower extremities without sural sparing [3]; relative uniform slowing of nerve conduction [4]; lesser degrees of conduction block and temporal dispersion [7, 10]. The organomegaly can be difficult to demonstrate on exam but is usually detected on imaging. VEGF is typically elevated, but can be normal in patients who received steroids as part of CIDP treatment. It is important to remember that skeletal x-ray surveys and PET scans can be negative in POEMS when the lesions are sclerotic, and therefore CT scan remains the screening test of choice when suspecting POEMS [12, 13]. Importantly, even on CT scans, bone lesions may be interpreted as benign. Very rarely, IFE and FLC are both negative in POEMS patients, and the monoclonal protein can only be demonstrated on 24-h urine testing or tissue biopsy. Finally, thrombocytosis is common in POEMS patients and can be a clue for this diagnosis in patients being investigated for demyelinating neuropathy.

CANOMAD is a rare disorder which tends to respond to similar treatment as CIDP, so in general the consequences of misdiagnosis are not major. Some patients with CANOMAD may experience episodes of acute worsening despite being on regular IVIG treatment. Patients should be suspected when they have IgM paraproteinemia and ophthalmoplegia [14].

Neurolymphomatosis can also sometimes be difficult to differentiate from CIDP [5]. Furthermore, neurolymphomatosis can initially respond to immunomodulatory treatments used for CIDP [5]. However, patients with neurolymphomatosis typically have pain, a feature rare in CIDP, and the disease is frequently focal early in the course of the disease. Furthermore, these patients tend to have systemic symptoms such as night sweat and weight loss [5].

The objective of this study was not to discuss all of the possible CIDP mimics. We wanted to report our own experience, specifically in patients who fulfilled definite ENFS/PNS criteria. Other groups have reported that hereditary transthyretin (hATTR) amyloidosis is frequently misdiagnosed as CIDP and occasionally fulfills ENFS/PNS criteria [8]. This has not been our experience. In our current cohort of 35 hATTR patients, 2 patients were misdiagnosed as CIDP but none fulfilled the ENFS/PNS criteria [15]. Other potential CIDP mimics reported include CMT4J (FIG4 mutation) [16], CMTX (Cx32 mutation) [17], mitochondrial neurogastrointestinal encephalomyopathy (MNGIE) [18], and additional diagnoses [19], but in general those have a different presentation and can usually be clinically differentiated from CIDP. One group of patients we did not include in this study are CIDP patients with antibodies to neurofascin and contactin. Some authors have suggested that patients with these antibodies should not be considered as a CIDP subtype but rather as an independent disease entity called CIDP-like chronic nodo-paranodopathy [20]. While that may be the case in the future, at this time we still include those patients under the CIDP umbrella.

## Conclusion

Most true CIDP mimics have pain, systemic symptoms, unique suggestive electrophysiological findings, and a serum monoclonal protein. Initial response to steroids or IVIG, over reliance on CSF and electrophysiology findings can all be misleading. Appropriate history and clinical examination, critical analysis of the demyelinating features on neurophysiology, and screening for monoclonal protein will lead to correct diagnosis in most cases.

## Data Availability

The datasets used and/or analyzed during the current study available from the corresponding author on reasonable request.

## Abbreviations

CIDP: chronic inflammatory demyelinating polyneuropathy
PLEX: plasma exchange
CSF: cerebral spinal fluid
ENFS/PNS: European Federation of Neurological Societies/Peripheral Nerve Society
POEMS: polyneuropathy, organomegaly, endocrinopathy, M protein and skin changes) syndrome
CANOMAD: Chronic Ataxic Neuropathy Ophthalmoplegia IgM paraprotein Cold Agglutinins Disialosyl antibodies
IVIG: intravenous immunoglobulin
VEGF: Vascular endothelial growth factor
CMT: Charcot Marie Tooth
MNGIE: mitochondrial neurogastrointestinal encephalomyopathy
TLI: terminal latency index

## References

[1] Van den Bergh PY, Hadden RD, Bouche P, Cornblath DR, Hahn A, Illa I (2010). European Federation of Neurological Societies/peripheral nerve society guideline on management of chronic inflammatory demyelinating polyradiculoneuropathy: report of a joint task force of the European Federation of Neurological Societies and the peripheral nerve society - first revision. Eur J Neurol.

[2] Tackenberg B, Lünemann JD, Steinbrecher A, Rothenfusser-Korber E, Sailer M, Brück W (2007). Classifications and treatment responses in chronic immune-mediated demyelinating polyneuropathy. Neurology..

[3] Allen JA, Lewis RA (2015). CIDP diagnostic pitfalls and perception of treatment benefit. Neurology..

[4] Kaplan A, Brannagan TH (2017). Evaluation of patients with refractory chronic inflammatory demyelinating polyneuropathy. Muscle Nerve.

[5] Tomita M, Koike H, Kawagashira Y, Iijima M, Adachi H, Taguchi J (2013). Clinicopathological features of neuropathy associated with lymphoma. Brain..

[6] Lahoria R, Karam C, Dispenzieri A, Dyck PJ (2013). Clinical reasoning: a 40-year-old man with CIDP-like illness resistant to treatment. Neurology..

[7] Nasu S, Misawa S, Sekiguchi Y, Shibuya K, Kanai K, Fujimaki Y (2012). Different neurological and physiological profiles in POEMS syndrome and chronic inflammatory demyelinating polyneuropathy. J Neurol Neurosurg Psychiatry.

[8] Cortese A, Vegezzi E, Lozza A, Alfonsi E, Montini A, Moglia A (2017). Diagnostic challenges in hereditary transthyretin amyloidosis with polyneuropathy: avoiding misdiagnosis of a treatable hereditary neuropathy. J Neurol Neurosurg Psychiatry.

[9] Wang Q, Liu P, Ji L-L, Wu S, Feng G-D, Wang X (2019). Clinical and electrophysiological profiles in early recognition of polyneuropathy, organomegaly, endocrinopathy, M-protein, and skin changes syndrome. Chin Med J.

[10] Mauermann ML, Sorenson EJ, Dispenzieri A, Mandrekar J, Suarez GA, Dyck PJ (2012). Uniform demyelination and more severe axonal loss distinguish POEMS syndrome from CIDP. J Neurol Neurosurg Psychiatry.

[11] Allen JA, Ney J, Lewis RA (2018). Electrodiagnostic errors contribute to chronic inflammatory demyelinating polyneuropathy misdiagnosis. Muscle Nerve.

[12] Glazebrook K, Guerra Bonilla FL, Johnson A, Leng S, Dispenzieri A (2015). Computed tomography assessment of bone lesions in patients with POEMS syndrome. Eur Radiol.

[13] Shibuya K, Misawa S, Horikoshi T, Kanai K, Isose S, Nasu S (2011). Detection of bone lesions by CT in POEMS syndrome. Intern Med.

[14] Garcia-Santibanez R, Zaidman CM, Sommerville RB, Lopate G, Weihl CC, Pestronk A (2018). CANOMAD and other chronic ataxic neuropathies with disialosyl antibodies (CANDA). J Neurol.

[15] Karam C, Dimitrova D, Heitner SB (2020). Misdiagnosis of hATTR amyloidosis: a single US site experience. Amyloid..

[16] Hu B, McCollum M, Ravi V, Arpag S, Moiseev D, Castoro R (2018). Myelin abnormality in Charcot-Marie-tooth type 4J recapitulates features of acquired demyelination. Ann Neurol.

[17] Sakaguchi H, Yamashita S, Miura A, Hirahara T, Kimura E, Maeda Y (2011). A novel GJB1 frameshift mutation produces a transient CNS symptom of X-linked Charcot-Marie-tooth disease. J Neurol.

[18] Bedlack RS, Vu T, Hammans S, Sparr SA, Myers B, Morgenlander J (2004). MNGIE neuropathy: five cases mimicking chronic inflammatory demyelinating polyneuropathy. Muscle Nerve.

[19] Neligan A, Reilly MM, Lunn MP (2014). CIDP: mimics and chameleons. Pract Neurol.

[20] Tang L, Huang Q, Qin Z, Tang X. Distinguish CIDP with autoantibody from that without autoantibody: pathogenesis, histopathology, and clinical features. J Neurol. 2020. 10.1007/s00415-020-09823-2 32266541

